# Crystal structure and Hirshfeld surface analysis of 2,2′-{(1*E*,1′*E*)-[ethane-1,2-diylbis(aza­nylyl­idene)]bis­(methanylyl­idene)}bis­[4-(tri­fluoro­meth­oxy)phenol]copper(II) hydro­quinone hemisolvate

**DOI:** 10.1107/S2056989019014294

**Published:** 2019-10-29

**Authors:** Sevgi Kansiz, Seher Meral, Necmi Dege, Aysen Alaman Agar, Igor O. Fritsky

**Affiliations:** a Department of Physics, Faculty of Arts and Sciences, Ondokuz Mayıs University, 55139 Kurupelit, Samsun, Turkey; b Boyabat Vocational School, Sinop University, 57200 Sinop, Turkey; c Department of Chemistry, Faculty of Arts and Sciences, Ondokuz Mayıs University, 55139 Kurupelit, Samsun, Turkey; d Department of Chemistry, Taras Shevchenko National University of Kyiv, 64 Vladimirska Str., Kiev 01601, Ukraine

**Keywords:** crystal structure, copper (II), salen-type Schiff base, Hirshfeld surface

## Abstract

In the title com­plex, [Cu(C_18_H_12_F_6_N_2_O_4_)]·0.5C_6_H_6_O_2_, the Cu^II^ ion has a square-planar coordination geometry, being ligated by two N and two O atoms of the tetra­dentate open-chain Schiff base ligand 6,6′-{(1*E*,1′*E*)-[ethane-1,2-diylbis(aza­nylyl­idene)]bis­(methanylyl­idene)}bis­[2-(tri­fluoro­meth­oxy)phenol]. The crystal packing is stabilized by intra­molecular O—H⋯O and inter­molecular C—H⋯F, C—H⋯O and C—H⋯π hydrogen bonds. In addition, weak π–π inter­actions form a three-dimensional structure. Hirshfeld surface analysis and two-dimensional fingerprint plots were performed and created to analyze the inter­molecular inter­actions present in the crystal, indicating that the most important contributions for the crystal packing are from F⋯H/H⋯F (25.7%), H⋯H (23.5%) and C⋯H/H⋯C (12.6%) inter­actions.

## Abstract 2

The title structure has a square-planar coordination sphere around the copper(II) ion. In the crystal, mol­ecules are linked by weak C—H⋯O and C—H⋯π hydrogen bonds and very weak π-stacking inter­actions, forming a three-dimensional supra­molecular architecture.

## Chemical context   

Metal com­plexes of Schiff bases have different applications because of their different heteroatoms (N, S, Cl *etc.*), functional groups, π-electron density, isomer structures and easy synthesis (El-Samanody *et al.*, 2017 ▸). Metal com­plexes with less oxophilic character exhibit attractive properties, such as targeting catalysts in many polymerization reactions (Ng *et al.*, 2016 ▸). On the other hand, in nature, metal com­plexes are encountered in many reactions, such as binding to DNA or enzymes (Li *et al.*, 2010 ▸). For this reason, metal com­plexes are of increasing inter­est in the fields of medicine and chemical synthesis with attractive functional properties and stable structures. Salen-type Schiff bases [salen is *N*,*N*′-bis­(salicyl­idene)ethyl­enedi­amine] have been synthesized by many research groups from different di­amines and derivatives of benzaldehyde (Prushan *et al.*, 2007 ▸). In addition, salen-type Schiff bases derived from 2-hy­droxy-3-meth­oxy­benzaldehyde (also called *o*-vanillin) are very effective ligands for many metal ions due to the two different binding sites, because of the presence of the meth­oxy group near the –OH group (Andruh, 2015 ▸). Each transition metal has different biological properties depending on the geometry of the com­plex and the structure of the ligand, so the biological activity of a drug may be controlled by changing the metal ion or the chemical structure of the ligand. Recently, it was reported that synthesized Schiff bases indicate anti­bacterial properties, more pronounced in the case of metal com­plexes com­pared to the free Schiff bases (Wu *et al.*, 2011 ▸).

In this study, a salen-type Schiff base has been synthesized from 2-hy­droxy-5-(tri­fluoro­meth­oxy)benzaldehyde with ethyl­enedi­amine by a condensation reaction. The synthesized Schiff base was used as an *O*,*N*,*N*′,*O*′-type tetra­dentate ligand, and a copper(II) com­plex was obtained and the structure confirmed by single-crystal X-ray diffraction analysis. In this study, we describe the crystal structure and Hirshfeld surface analysis of the title com­pound, as determined by X-ray crystallographic analysis.
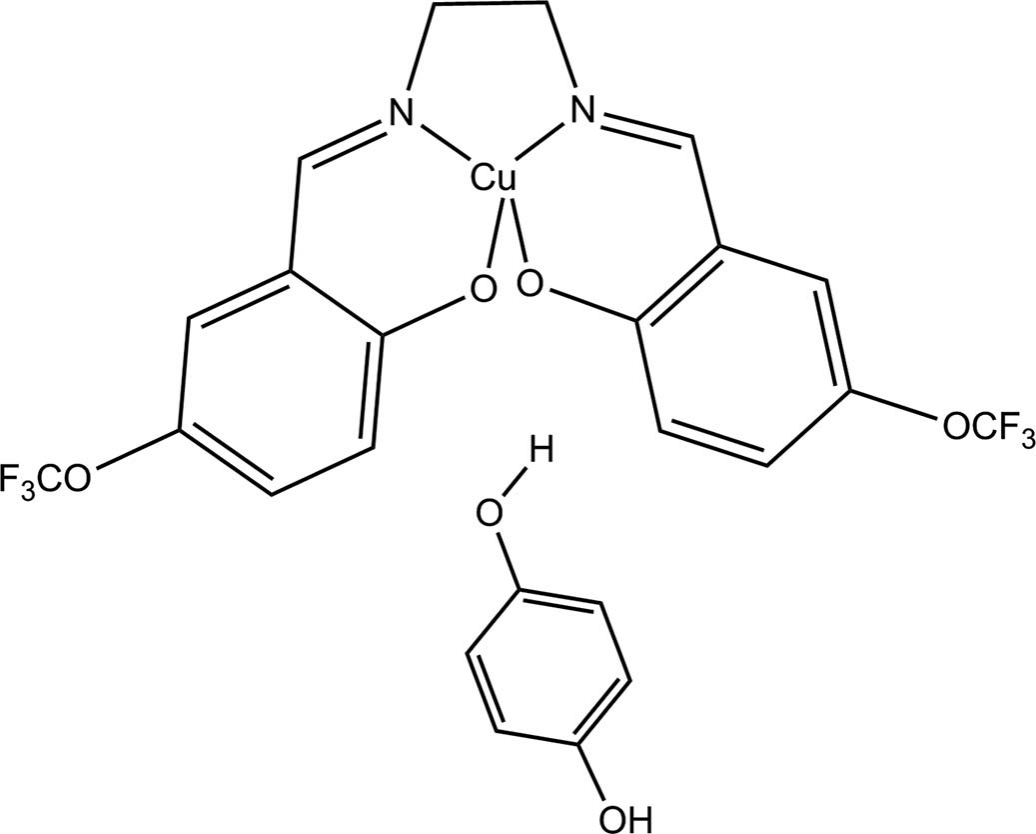


## Structural commentary   

**Figure 1:**
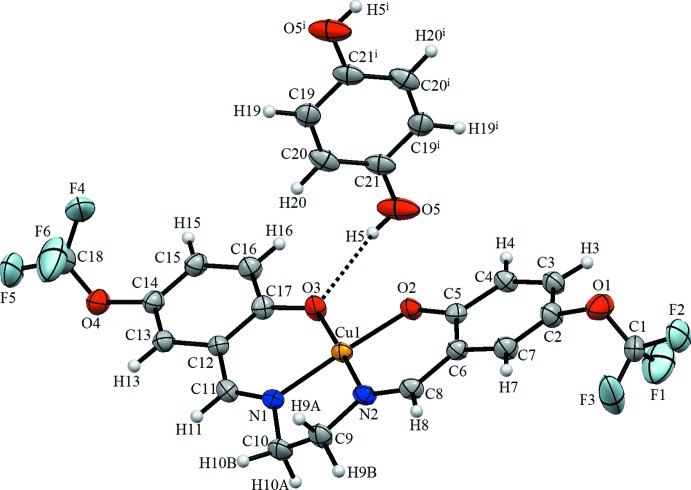
The mol­ecular structure of the title com­pound, with the atom labelling. Displacement ellipsoids are drawn at the 30% probability level. The dashed line indicates a hydrogen bond. [Symmetry code: (i) −*x*, −*y*, −*z* + 1.]

Fig. 1 ▸ illustrates the title metal com­plex formed by a Cu^II^ ion chelated by a doubly deprotonated tetra­dentate Schiff base ligand and a hydrogen-bonded mol­ecule of hydro­quinone. The Cu1 ion is coordinated by two imine N atoms (N6 and N7) and two phenoxo O atoms (O2 and O3) of the tetra­dentate Schiff base ligand 6,6′-{(1*E*,1′*E*)-[ethane-1,2-diylbis(aza­nylyl­idene)]bis­(methanylyl­idene)}bis­[2-(tri­fluoro­meth­oxy)phenol] (**L1**). The hydro­quinone mol­ecule is located on an inversion centre and is linked to neighbouring com­plex cations *via* O—H⋯O hydrogen bonds. The bond lengths Cu1—O2 and Cu1—O3 [1.883 (4) and 1.906 (4) Å, respectively] and Cu1—N1 and Cu1—N2 [1.929 (5) and 1.927 (5) Å, respectively] are close to the values observed for related copper(II) com­plexes reported in the literature (Şen *et al.*, 2017 ▸; Fritsky *et al.*, 2004 ▸; Strotmeyer *et al.*, 2003 ▸). Selected geometric parameters of the title com­pound are listed in Table 1 ▸.

## Supra­molecular features   

**Figure 2:**
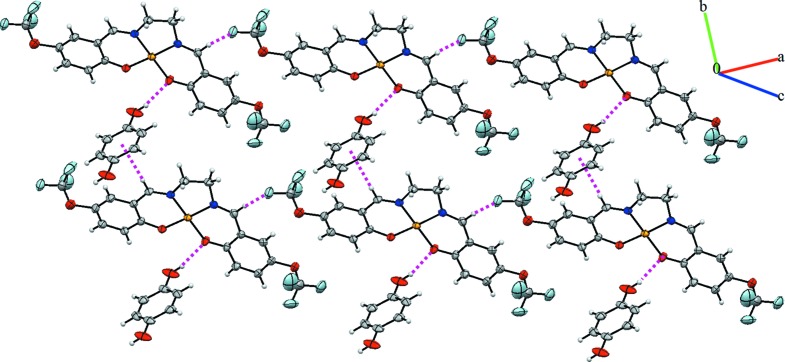
A view of the crystal packing of the title com­pound. Dashed lines denote inter­molecular O—H⋯O, C—H⋯F and C—H⋯π hydrogen bonds.

**Figure 3:**
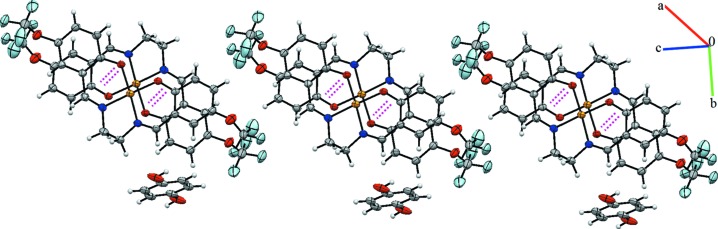
A view of the crystal packing of the title com­pound. The π–π inter­actions are shown as pink dashed lines. **[The direction of the unitcell parameters is missing. It might be better to show the unitcell outline]**

The crystal packing of the title com­pound is stabilized by inter­molecular C—H⋯O, C—H⋯F and C—H⋯*Cg*1 (*Cg*1 is the centroid of the C19–C21/C19^i^–C21^i^ ring) hydrogen bonds (Table 2 ▸ and Fig. 2 ▸). In addition, weak π–π inter­actions connect the mol­ecules into a three-dimensional supra­molecular architecture (Fig. 3 ▸). The *Cg*2⋯*Cg*3 distance is 3.507 (2) Å, where *Cg*2 and *Cg*3 are the centroids of the Cu1/O2/C5/C6/C8/N2 and Cu1/O3/C17/C12/C11/N1 rings, respectively.

## Hirshfeld surface analysis   

**Figure 4:**
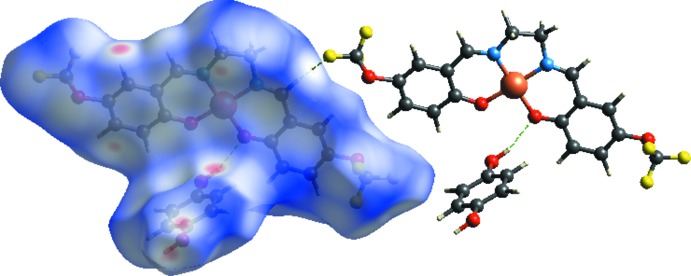
The *d*_norm_-mapped Hirshfeld surface for visualizing the inter­molecular contacts of the title com­pound.

The Hirshfeld surface analysis (Spackman & Jayatilaka, 2009 ▸) and the associated two-dimensional fingerprint plots were performed and created with *CrystalExplorer17* (Turner *et al.*, 2017 ▸). The Hirshfeld surface was mapped with *d*_norm_ (Fig. 4 ▸). The view of surface were obtained in the range −0.4385 to 1.6105 a.u. (*d*_norm_). The blue, white and red colour conventions used for the *d*_norm_-mapped Hirshfeld surfaces recognize the inter­atomic contacts as longer, at van der Waals separations and short inter­atomic contacts, respectively.

**Figure 5:**
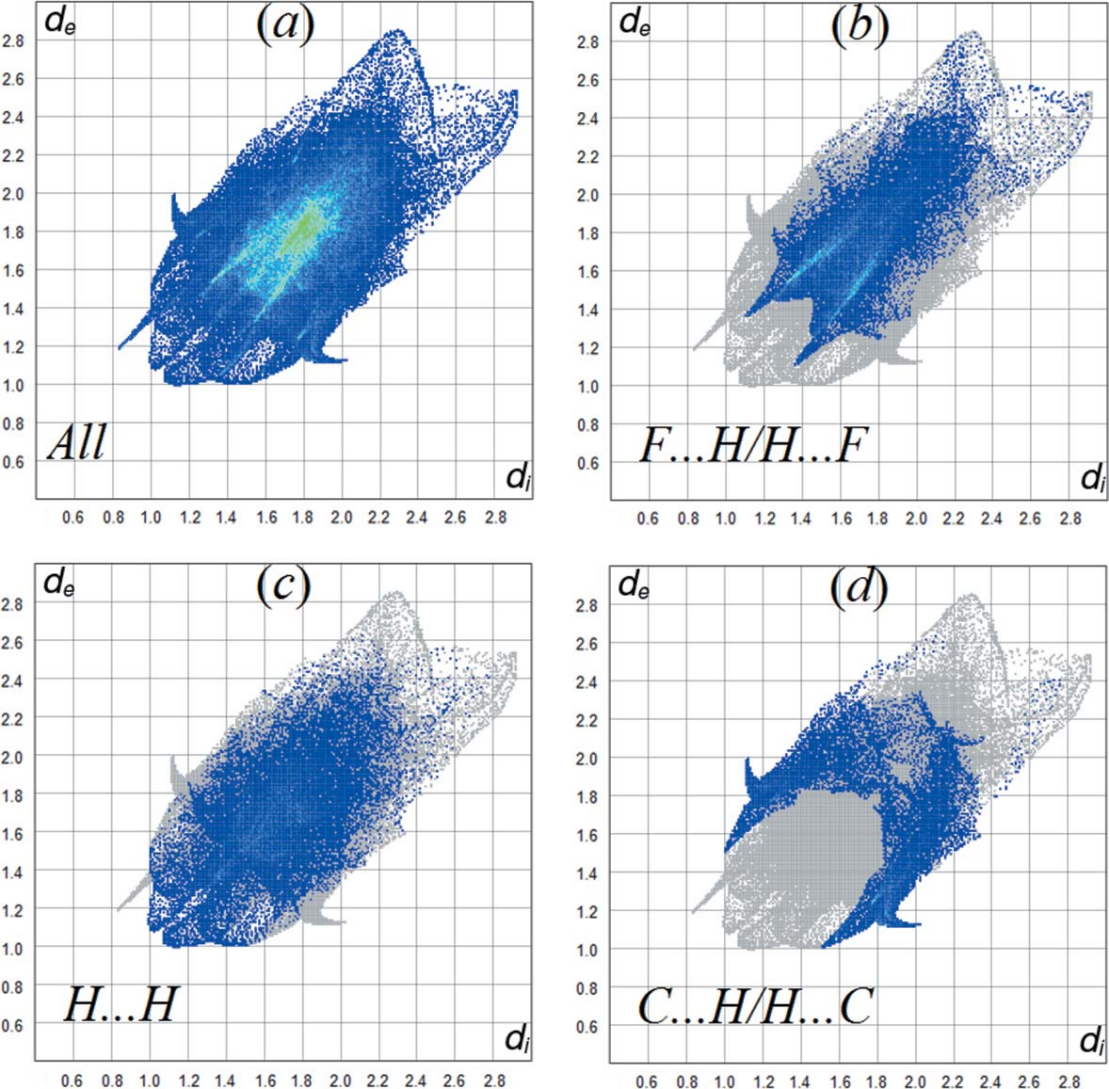
Two-dimensional fingerprint plots of the title com­pound.

A fingerprint plot delineated into specific inter­atomic contacts contains information related to specific inter­molecular inter­actions. The blue colour refers to the frequency of occurrence of the (*d*_i_, *d*_e_) pair with the full fingerprint plot outlined in gray. Fig. 5 ▸(*a*) shows the two-dimensional fingerprint plot of the sum of the contacts contributing to the Hirshfeld surface represented in normal mode. The most significant contribution to the Hirshfeld surface is from F⋯H/H⋯F contacts (25.7%) (Fig. 5 ▸ *b*). Here, H⋯H interactions are only the second most significant contribution to the total Hirshfeld surface (23.5%). In addition, C⋯H/H⋯C and O⋯H/H⋯O contacts contribute 12.6 and 11.2% to the Hirshfeld surface, respectively.

## Database survey   

A search of the Cambridge Structural Database (CSD, Version 5.40, update of February 2019; Groom *et al.*, 2016 ▸) related to the title com­plex revealed six hits. These structures are Cu(5-hexyl­oxySalen)·CHCl_3_ (FAGLOP; Paschke *et al.*, 2002 ▸), C_30_H_54_Cu_2_F_12_N_10_O_2_P_2_ (ICUHEU; Margraf *et al.*, 2006 ▸), C_38_H_44_Cu_2_N_4_O_10_ (PIFKOE01; Liu, 2016 ▸), C_36_H_36_Cu_2_N_4_O_8_·2CH_4_O (PIFKOE02; Zhang, 2016 ▸), C_18_H_18_CuN_2_O_4_·1.5H_2_O (QARPAB; Yao *et al.*, 2005 ▸) and C_18_H_18_CuN_2_O_4_ (XOZ­ZUH; Atria *et al.*, 2002 ▸). All of these structures have square-planar environments, as in the title copper(II) com­plex. The Cu—O and Cu—N bond lengths range from *ca* 1.898 to 1.915 Å and from *ca* 1.936 to 2.271 Å, respectively. In the title com­plex, the Cu—N bond lengths [1.927 (5) and 1.929 (5) Å] fall within these limits. While the Cu1—O3 and C1—O2 bond length [1.906 (4) and 1.883 (4) Å, respectively] are within and close to these limits, respectively, the Cu1—O2 bond length is outside these limits, with a shorter value of 1.883 (4) Å.

## Synthesis and crystallization   

**Figure 6:**
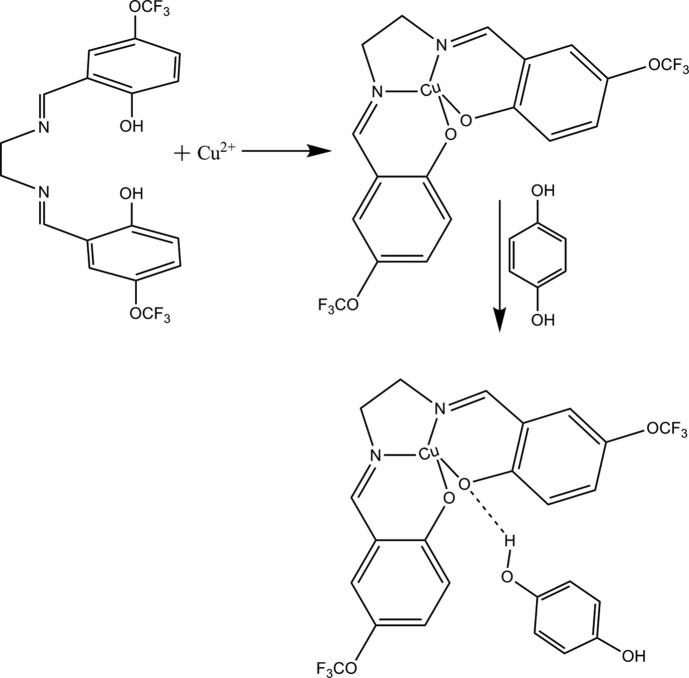
The synthesis of the title com­pound.

2,2′-{(1*E*,1′*E*)-[Ethane-1,2-diylbis(aza­nylyl­idene)]bis­(methan­ylyl­idene)}bis­[4-(tri­fluoro­meth­oxy)phenol] (H_2_ **L1**) was syn­thesized by condensation of 2-hy­droxy-5-(tri­­fluoro­meth­oxy)­benzaldehyde (0.0095 mmol) and 1,2-ethane­diamine (0.0095 mmol) in ethanol under reflux for about 18 h. The yellow product was washed with ether and dried at room temperature. 0.0080 mmol H_2_ **L1** was dissolved in 20 ml ethanol and 0.0080 mmol Cu(CH_3_COO)_2_·H_2_O was dissolved in 20 ml ethanol. The metal solution was added dropwise to the Schiff base solution and the resulting solution refluxed for about 6 h. The product (Cu**L1**) was washed with toluene and crystallized from ethanol at room temperature. 2,2′-{(1*E*,1′*E*)-[Ethane-1,2-diylbis(aza­nylyl­idene)]bis­(methanylyl­idene)}bis­[4-(tri­fluoro­meth­oxy)phenol]copper(II) hydro­quinone hemisolvate was obtained even after 0.0040 mmol hydro­quinone was added to 0.0040 mmol Cu**L1** in 20 ml ethanol and refluxed for about 6 h. A purple crystal suitable for X-ray diffraction analysis was obtained from the reaction (m.p. 568 K; yield 80%) (Fig. 6 ▸).

## Refinement   

Crystal data, data collection and structure refinement details are summarized in Table 3 ▸. All H atoms were fixed geometrically and treated as riding, with C—H = 0.97 Å and *U*_iso_(H) = 1.2*U*_eq_(C) for methyl­ene, C—H = 0.93 Å and *U*_iso_(H) = 1.2*U*_eq_(C) for aromatic, C—H = 0.93 Å and *U*_iso_(H) = 1.2*U*_eq_(C) for methine, and O—H = 0.82 Å and *U*_iso_(H) = 1.5*U*_eq_(O) for hy­droxy H atoms.

## Supplementary Material

Crystal structure: contains datablock(s) I, global. DOI: 10.1107/S2056989019014294/lh5932sup1.cif

Structure factors: contains datablock(s) I. DOI: 10.1107/S2056989019014294/lh5932Isup2.hkl

CCDC references: 1900670, 1900670

Additional supporting information:  crystallographic information; 3D view; checkCIF report

## Figures and Tables

**Table 1:** Selected geometric parameters (Å, °)

Cu1—O2	1.883 (4)	N3—C10	1.455 (8)
Cu1—O3	1.906 (4)	N2—C8	1.275 (8)
Cu1—N2	1.927 (5)	O4—C18	1.269 (12)
Cu1—N3	1.929 (5)	O1—C1	1.267 (9)
O2—C5	1.309 (7)	F2—C1	1.271 (9)
O3—C17	1.317 (7)	F4—C18	1.265 (11)
			
O2—Cu1—O3	87.54 (17)	O2—Cu1—N3	177.82 (18)
O2—Cu1—N2	94.40 (18)	O3—Cu1—N3	93.9 (2)
O3—Cu1—N2	176.2 (2)	N2—Cu1—N3	84.3 (2)

**Table 2:** Hydrogen-bond geometry (Å, °) *Cg*1 is the centroid of the C19–C21/C19^i^–C21^i^ ring.

*D*—H⋯*A*	*D*—H	H⋯*A*	*D*⋯*A*	*D*—H⋯*A*
O5—H5⋯O3	0.82	2.20	2.993 (8)	165
C11—H11⋯F2^i^	0.93	2.63	3.513 (8)	159
C10—H10*A*⋯O5^ii^	0.97	2.53	3.469 (11)	162
C15—H15⋯O5^iii^	0.93	2.55	3.345 (9)	144
C8—H8⋯*Cg*1^iv^	0.93	2.82	3.740 (8)	173

Symmetry codes: (i) 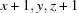; (ii) 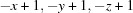; (iii) 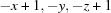; (iv) 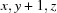.

**Table 3:** Experimental details

Crystal data	
Chemical formula	[Cu(C_18_H_12_F_6_N_2_O_4_)]·0.5C_6_H_6_O_2_
*M*_r_	552.89
Crystal system, space group	Triclinic, *P* 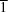
Temperature (K)	296
*a*, *b*, *c* (Å)	9.3167 (10), 10.0363 (10), 11.8052 (13)
α, β, γ (°)	92.633 (9), 97.310 (9), 98.670 (9)
*V* (Å^3^)	1080.0 (2)
*Z*	2
Radiation type	Mo *K*α
μ (mm^−1^)	1.10
Crystal size (mm)	0.57 × 0.25 × 0.06

Data collection	
Diffractometer	Stoe *IPDS* 2
Absorption correction	Integration (*X-RED32*; Stoe & Cie, 2002 ▸)
*T*_min_, *T*_max_	0.695, 0.944
No. of measured, independent and observed [*I* > 2σ(*I*)] reflections	9277, 4105, 2818
*R*_int_	0.105
(sin θ/λ)_max_ (Å^−1^)	0.617

Refinement	
*R*[*F* ^2^ > 2σ(*F* ^2^)], *wR*(*F* ^2^), *S*	0.067, 0.217, 1.08
No. of reflections	4105
No. of parameters	317
H-atom treatment	H-atom parameters constrained
Δρ_max_, Δρ_min_ (e Å^−3^)	0.88, −0.43

Computer programs: *X-AREA* (Stoe & Cie, 2002 ▸), *X-RED* (Stoe & Cie, 2002 ▸), *SHELXT2017* (Sheldrick, 2015*a* ▸), *SHELXL2017* (Sheldrick, 2015*b* ▸), *PLATON* (Spek, 2009 ▸) and *WinGX* (Farrugia, 2012 ▸).

## supplementary crystallographic information

### Crystal data

**Table:**

[Cu(C_18_H_12_F_6_N_2_O_4_)]·0.5C_6_H_6_O_2_	*Z* = 2
*M**_r_* = 552.89	*F*(000) = 556
Triclinic, *P*1	*D*_x_ = 1.700 Mg m^−^^3^
*a* = 9.3167 (10) Å	Mo *K*α radiation, λ = 0.71073 Å
*b* = 10.0363 (10) Å	Cell parameters from 9488 reflections
*c* = 11.8052 (13) Å	θ = 2.1–31.6°
α = 92.633 (9)°	µ = 1.10 mm^−^^1^
β = 97.310 (9)°	*T* = 296 K
γ = 98.670 (9)°	Stick, orange
*V* = 1080.0 (2) Å^3^	0.57 × 0.25 × 0.06 mm

### Data collection

**Table:**

Stoe IPDS 2 diffractometer	4105 independent reflections
Radiation source: sealed X-ray tube, 12 x 0.4 mm long-fine focus	2818 reflections with *I* > 2σ(*I*)
Detector resolution: 6.67 pixels mm^-1^	*R*_int_ = 0.105
rotation method scans	θ_max_ = 26.0°, θ_min_ = 2.1°
Absorption correction: integration (X-RED32; Stoe & Cie, 2002)	*h* = −11→11
*T*_min_ = 0.695, *T*_max_ = 0.944	*k* = −11→12
9277 measured reflections	*l* = −14→14

### Refinement

**Table:**

Refinement on *F*^2^	0 restraints
Least-squares matrix: full	Hydrogen site location: inferred from neighbouring sites
*R*[*F*^2^ > 2σ(*F*^2^)] = 0.067	H-atom parameters constrained
*wR*(*F*^2^) = 0.217	*w* = 1/[σ^2^(*F*_o_^2^) + (0.1271*P*)^2^] where *P* = (*F*_o_^2^ + 2*F*_c_^2^)/3
*S* = 1.08	(Δ/σ)_max_ < 0.001
4105 reflections	Δρ_max_ = 0.88 e Å^−^^3^
317 parameters	Δρ_min_ = −0.43 e Å^−^^3^

### Special details

**Table:**

Geometry. All esds (except the esd in the dihedral angle between two l.s. planes) are estimated using the full covariance matrix. The cell esds are taken into account individually in the estimation of esds in distances, angles and torsion angles; correlations between esds in cell parameters are only used when they are defined by crystal symmetry. An approximate (isotropic) treatment of cell esds is used for estimating esds involving l.s. planes.

### Fractional atomic coordinates and isotropic or equivalent isotropic displacement parameters (Å^2^)

**Table:**

	*x*	*y*	*z*	*U*_iso_*/*U*_eq_	
Cu1	0.34081 (8)	0.48948 (6)	0.55458 (5)	0.0543 (3)	
O2	0.2235 (5)	0.4083 (4)	0.4202 (3)	0.0623 (10)	
O3	0.4268 (5)	0.3290 (4)	0.5642 (3)	0.0725 (12)	
N3	0.4629 (6)	0.5787 (5)	0.6891 (4)	0.0615 (12)	
N2	0.2498 (5)	0.6496 (4)	0.5547 (4)	0.0599 (11)	
O4	0.8058 (6)	0.1792 (5)	0.9159 (5)	0.0941 (16)	
O1	−0.2353 (6)	0.5831 (6)	0.1519 (6)	0.1072 (19)	
F2	−0.3405 (7)	0.6958 (6)	0.0275 (5)	0.1250 (19)	
C5	0.1234 (6)	0.4598 (5)	0.3559 (5)	0.0562 (12)	
C17	0.5157 (6)	0.2963 (6)	0.6508 (5)	0.0594 (13)	
C12	0.5756 (7)	0.3861 (6)	0.7474 (5)	0.0607 (14)	
C13	0.6693 (7)	0.3408 (6)	0.8348 (5)	0.0656 (14)	
H13	0.708436	0.398326	0.898891	0.079*	
C11	0.5502 (7)	0.5230 (6)	0.7574 (5)	0.0652 (15)	
H11	0.602434	0.576870	0.819803	0.078*	
C4	0.0563 (7)	0.3889 (6)	0.2529 (5)	0.0640 (14)	
H4	0.086152	0.308254	0.231398	0.077*	
O5	0.2281 (8)	0.1271 (6)	0.3981 (7)	0.133 (3)	
H5	0.291517	0.170047	0.446142	0.199*	
C6	0.0774 (7)	0.5844 (5)	0.3848 (5)	0.0600 (13)	
C8	0.1441 (7)	0.6725 (6)	0.4822 (5)	0.0637 (14)	
H8	0.107514	0.752591	0.493445	0.076*	
C14	0.7035 (7)	0.2153 (6)	0.8275 (6)	0.0701 (16)	
C20	0.1237 (8)	0.0623 (7)	0.5681 (7)	0.083 (2)	
H20	0.206608	0.107143	0.614594	0.099*	
F5	0.8619 (9)	0.0840 (7)	1.0705 (6)	0.165 (3)	
C2	−0.1012 (8)	0.5510 (7)	0.2184 (6)	0.0809 (19)	
C16	0.5574 (8)	0.1686 (6)	0.6466 (6)	0.0716 (16)	
H16	0.521840	0.109651	0.582720	0.086*	
C1	−0.2238 (10)	0.6854 (8)	0.0927 (6)	0.089 (2)	
F4	0.6940 (12)	−0.0204 (7)	0.9451 (6)	0.213 (5)	
C9	0.3081 (9)	0.7467 (6)	0.6522 (6)	0.0793 (19)	
H9A	0.243130	0.737135	0.710360	0.095*	
H9B	0.313865	0.838005	0.627497	0.095*	
C3	−0.0517 (8)	0.4351 (7)	0.1834 (6)	0.0784 (19)	
H3	−0.091257	0.389063	0.113585	0.094*	
F3	−0.1763 (13)	0.7969 (6)	0.1498 (6)	0.220 (5)	
C19	0.0109 (9)	−0.0032 (6)	0.6171 (6)	0.084 (2)	
H19	0.019589	−0.007714	0.696149	0.101*	
C7	−0.0395 (8)	0.6257 (7)	0.3149 (6)	0.0793 (19)	
H7	−0.074097	0.704473	0.335183	0.095*	
C10	0.4551 (9)	0.7224 (6)	0.7003 (6)	0.086 (2)	
H10A	0.528371	0.772004	0.660172	0.104*	
H10B	0.475229	0.754184	0.780449	0.104*	
F1	−0.1335 (8)	0.6744 (10)	0.0196 (7)	0.179 (3)	
C21	0.1175 (9)	0.0633 (6)	0.4511 (7)	0.085 (2)	
C15	0.6497 (7)	0.1268 (7)	0.7341 (6)	0.0730 (16)	
H15	0.675021	0.040716	0.729992	0.088*	
F6	0.6605 (12)	0.1453 (14)	1.0406 (8)	0.215 (5)	
C18	0.7608 (15)	0.0945 (11)	0.9851 (10)	0.122 (4)	

### Atomic displacement parameters (Å^2^)

**Table:**

	*U*^11^	*U*^22^	*U*^33^	*U*^12^	*U*^13^	*U*^23^
Cu1	0.0612 (5)	0.0452 (4)	0.0562 (4)	0.0114 (3)	0.0058 (3)	−0.0023 (2)
O2	0.073 (3)	0.0482 (19)	0.064 (2)	0.0178 (17)	−0.0043 (18)	−0.0075 (16)
O3	0.094 (3)	0.059 (2)	0.064 (2)	0.037 (2)	−0.012 (2)	−0.0105 (18)
N3	0.068 (3)	0.052 (2)	0.060 (3)	0.002 (2)	0.003 (2)	−0.0008 (19)
N2	0.062 (3)	0.046 (2)	0.071 (3)	0.012 (2)	0.004 (2)	−0.006 (2)
O4	0.088 (3)	0.082 (3)	0.106 (4)	0.015 (3)	−0.022 (3)	0.029 (3)
O1	0.091 (4)	0.096 (4)	0.132 (5)	0.013 (3)	−0.005 (3)	0.038 (4)
F2	0.130 (4)	0.118 (4)	0.120 (4)	0.043 (3)	−0.042 (3)	0.018 (3)
C5	0.054 (3)	0.054 (3)	0.062 (3)	0.011 (2)	0.009 (2)	0.004 (2)
C17	0.054 (3)	0.062 (3)	0.068 (3)	0.026 (3)	0.010 (3)	0.003 (3)
C12	0.065 (4)	0.054 (3)	0.060 (3)	0.003 (3)	0.002 (3)	0.007 (2)
C13	0.065 (4)	0.059 (3)	0.071 (3)	0.008 (3)	0.003 (3)	0.006 (3)
C11	0.061 (3)	0.068 (4)	0.062 (3)	0.006 (3)	0.000 (3)	−0.003 (3)
C4	0.061 (3)	0.056 (3)	0.074 (3)	0.018 (3)	0.002 (3)	−0.008 (3)
O5	0.146 (6)	0.068 (3)	0.188 (7)	−0.016 (3)	0.094 (6)	−0.049 (4)
C6	0.062 (3)	0.049 (3)	0.070 (3)	0.016 (2)	0.002 (3)	0.005 (2)
C8	0.069 (4)	0.048 (3)	0.076 (4)	0.021 (3)	0.008 (3)	−0.004 (2)
C14	0.067 (4)	0.063 (3)	0.078 (4)	0.012 (3)	−0.004 (3)	0.018 (3)
C20	0.070 (4)	0.057 (4)	0.119 (6)	0.017 (3)	0.004 (4)	−0.029 (4)
F5	0.219 (7)	0.133 (5)	0.126 (4)	0.032 (5)	−0.067 (5)	0.044 (4)
C2	0.081 (5)	0.068 (4)	0.090 (4)	0.025 (3)	−0.018 (4)	0.007 (3)
C16	0.078 (4)	0.061 (3)	0.077 (4)	0.025 (3)	0.000 (3)	−0.003 (3)
C1	0.105 (6)	0.093 (5)	0.060 (4)	0.004 (4)	−0.007 (4)	0.014 (4)
F4	0.351 (12)	0.082 (4)	0.151 (6)	−0.054 (5)	−0.078 (7)	0.038 (4)
C9	0.102 (5)	0.054 (3)	0.077 (4)	0.020 (3)	−0.008 (4)	−0.013 (3)
C3	0.090 (5)	0.063 (4)	0.075 (4)	0.011 (3)	−0.010 (3)	−0.005 (3)
F3	0.390 (13)	0.087 (4)	0.149 (5)	0.083 (6)	−0.138 (7)	−0.026 (4)
C19	0.117 (6)	0.055 (3)	0.081 (4)	0.022 (4)	0.009 (4)	−0.008 (3)
C7	0.088 (5)	0.059 (3)	0.090 (4)	0.023 (3)	−0.006 (4)	0.001 (3)
C10	0.112 (6)	0.051 (3)	0.085 (4)	0.002 (3)	−0.012 (4)	−0.008 (3)
F1	0.151 (6)	0.259 (10)	0.144 (6)	0.038 (6)	0.057 (5)	0.058 (6)
C21	0.093 (5)	0.047 (3)	0.114 (6)	0.007 (3)	0.026 (4)	−0.021 (3)
C15	0.076 (4)	0.061 (3)	0.085 (4)	0.021 (3)	0.003 (3)	0.016 (3)
F6	0.202 (9)	0.316 (14)	0.137 (6)	0.034 (9)	0.043 (6)	0.097 (7)
C18	0.149 (10)	0.098 (7)	0.101 (6)	−0.003 (6)	−0.028 (7)	0.026 (5)

### Geometric parameters (Å, º)

**Table:**

Cu1—O2	1.883 (4)	O5—H5	0.8200
Cu1—O3	1.906 (4)	C6—C7	1.407 (9)
Cu1—N2	1.927 (5)	C6—C8	1.431 (8)
Cu1—N3	1.929 (5)	C8—H8	0.9300
O2—C5	1.309 (7)	C14—C15	1.375 (9)
O3—C17	1.317 (7)	C20—C19	1.361 (11)
N3—C11	1.279 (8)	C20—C21	1.375 (11)
N3—C10	1.455 (8)	C20—H20	0.9300
N2—C8	1.275 (8)	F5—C18	1.309 (11)
N2—C9	1.465 (7)	C2—C7	1.348 (10)
O4—C18	1.269 (12)	C2—C3	1.379 (10)
O4—C14	1.419 (7)	C16—C15	1.381 (9)
O1—C1	1.267 (9)	C16—H16	0.9300
O1—C2	1.475 (8)	C1—F3	1.268 (9)
F2—C1	1.271 (9)	C1—F1	1.292 (10)
C5—C4	1.403 (8)	F4—C18	1.265 (11)
C5—C6	1.423 (8)	C9—C10	1.474 (11)
C17—C16	1.395 (8)	C9—H9A	0.9700
C17—C12	1.420 (8)	C9—H9B	0.9700
C12—C13	1.403 (8)	C3—H3	0.9300
C12—C11	1.431 (9)	C19—C21^i^	1.392 (11)
C13—C14	1.346 (9)	C19—H19	0.9300
C13—H13	0.9300	C7—H7	0.9300
C11—H11	0.9300	C10—H10A	0.9700
C4—C3	1.366 (9)	C10—H10B	0.9700
C4—H4	0.9300	C15—H15	0.9300
O5—C21	1.366 (9)	F6—C18	1.353 (15)
			
O2—Cu1—O3	87.54 (17)	C21—C20—H20	119.4
O2—Cu1—N2	94.40 (18)	C7—C2—C3	122.2 (6)
O3—Cu1—N2	176.2 (2)	C7—C2—O1	120.4 (7)
O2—Cu1—N3	177.82 (18)	C3—C2—O1	117.0 (6)
O3—Cu1—N3	93.9 (2)	C15—C16—C17	122.2 (6)
N2—Cu1—N3	84.3 (2)	C15—C16—H16	118.9
C5—O2—Cu1	127.1 (3)	C17—C16—H16	118.9
C17—O3—Cu1	127.0 (4)	F3—C1—O1	114.8 (7)
C11—N3—C10	122.4 (5)	F3—C1—F2	109.4 (8)
C11—N3—Cu1	125.0 (4)	O1—C1—F2	113.7 (7)
C10—N3—Cu1	112.3 (4)	F3—C1—F1	105.7 (9)
C8—N2—C9	120.3 (5)	O1—C1—F1	110.6 (9)
C8—N2—Cu1	125.9 (4)	F2—C1—F1	101.6 (7)
C9—N2—Cu1	113.8 (4)	N2—C9—C10	109.6 (6)
C18—O4—C14	118.9 (7)	N2—C9—H9A	109.7
C1—O1—C2	118.1 (6)	C10—C9—H9A	109.7
O2—C5—C4	118.9 (5)	N2—C9—H9B	109.7
O2—C5—C6	123.6 (5)	C10—C9—H9B	109.7
C4—C5—C6	117.5 (5)	H9A—C9—H9B	108.2
O3—C17—C16	118.8 (5)	C4—C3—C2	119.0 (6)
O3—C17—C12	123.4 (5)	C4—C3—H3	120.5
C16—C17—C12	117.8 (5)	C2—C3—H3	120.5
C13—C12—C17	118.7 (6)	C20—C19—C21^i^	119.9 (7)
C13—C12—C11	118.3 (5)	C20—C19—H19	120.0
C17—C12—C11	123.0 (5)	C21^i^—C19—H19	120.0
C14—C13—C12	121.1 (6)	C2—C7—C6	119.8 (6)
C14—C13—H13	119.5	C2—C7—H7	120.1
C12—C13—H13	119.5	C6—C7—H7	120.1
N3—C11—C12	126.5 (5)	N3—C10—C9	109.9 (5)
N3—C11—H11	116.7	N3—C10—H10A	109.7
C12—C11—H11	116.7	C9—C10—H10A	109.7
C3—C4—C5	121.9 (6)	N3—C10—H10B	109.7
C3—C4—H4	119.1	C9—C10—H10B	109.7
C5—C4—H4	119.1	H10A—C10—H10B	108.2
C21—O5—H5	109.5	O5—C21—C20	123.4 (7)
C7—C6—C5	119.3 (5)	O5—C21—C19^i^	117.9 (8)
C7—C6—C8	117.1 (5)	C20—C21—C19^i^	118.7 (7)
C5—C6—C8	123.6 (5)	C14—C15—C16	118.5 (6)
N2—C8—C6	125.1 (5)	C14—C15—H15	120.7
N2—C8—H8	117.5	C16—C15—H15	120.7
C6—C8—H8	117.5	F4—C18—O4	118.6 (10)
C13—C14—C15	121.7 (6)	F4—C18—F5	111.2 (9)
C13—C14—O4	117.9 (6)	O4—C18—F5	112.4 (10)
C15—C14—O4	120.2 (6)	F4—C18—F6	103.2 (13)
C19—C20—C21	121.3 (7)	O4—C18—F6	108.5 (10)
C19—C20—H20	119.4	F5—C18—F6	101.1 (11)
			
O3—Cu1—O2—C5	179.3 (5)	C18—O4—C14—C15	−74.5 (11)
N2—Cu1—O2—C5	2.6 (5)	C1—O1—C2—C7	74.4 (11)
Cu1—O2—C5—C4	174.2 (4)	C1—O1—C2—C3	−111.7 (9)
Cu1—O2—C5—C6	−6.2 (8)	O3—C17—C16—C15	−179.8 (6)
Cu1—O3—C17—C16	174.3 (5)	C12—C17—C16—C15	2.2 (10)
Cu1—O3—C17—C12	−7.8 (9)	C2—O1—C1—F3	−61.3 (13)
O3—C17—C12—C13	−180.0 (6)	C2—O1—C1—F2	171.7 (7)
C16—C17—C12—C13	−2.0 (9)	C2—O1—C1—F1	58.2 (10)
O3—C17—C12—C11	−2.9 (10)	C8—N2—C9—C10	−161.6 (6)
C16—C17—C12—C11	175.0 (6)	Cu1—N2—C9—C10	21.2 (7)
C17—C12—C13—C14	0.6 (10)	C5—C4—C3—C2	−3.4 (11)
C11—C12—C13—C14	−176.5 (6)	C7—C2—C3—C4	4.9 (12)
C10—N3—C11—C12	−173.9 (7)	O1—C2—C3—C4	−168.9 (6)
Cu1—N3—C11—C12	0.6 (10)	C21—C20—C19—C21^i^	−4.1 (12)
C13—C12—C11—N3	−176.2 (6)	C3—C2—C7—C6	−1.4 (12)
C17—C12—C11—N3	6.8 (10)	O1—C2—C7—C6	172.2 (6)
O2—C5—C4—C3	178.2 (6)	C5—C6—C7—C2	−3.6 (10)
C6—C5—C4—C3	−1.5 (9)	C8—C6—C7—C2	176.2 (7)
O2—C5—C6—C7	−174.7 (6)	C11—N3—C10—C9	−153.1 (6)
C4—C5—C6—C7	4.9 (9)	Cu1—N3—C10—C9	31.8 (8)
O2—C5—C6—C8	5.5 (9)	N2—C9—C10—N3	−33.6 (9)
C4—C5—C6—C8	−174.8 (6)	C19—C20—C21—O5	−179.7 (7)
C9—N2—C8—C6	−179.2 (6)	C19—C20—C21—C19^i^	4.1 (12)
Cu1—N2—C8—C6	−2.3 (9)	C13—C14—C15—C16	−0.6 (11)
C7—C6—C8—N2	179.2 (6)	O4—C14—C15—C16	−176.1 (6)
C5—C6—C8—N2	−1.0 (10)	C17—C16—C15—C14	−0.9 (11)
C12—C13—C14—C15	0.7 (11)	C14—O4—C18—F4	56.4 (17)
C12—C13—C14—O4	176.3 (6)	C14—O4—C18—F5	−171.6 (9)
C18—O4—C14—C13	109.8 (10)	C14—O4—C18—F6	−60.7 (11)

Symmetry code: (i) −*x*, −*y*, −*z*+1.

### Hydrogen-bond geometry (Å, º)

**Table:**

*D*—H···*A*	*D*—H	H···*A*	*D*···*A*	*D*—H···*A*
O5—H5···O3	0.82	2.20	2.993 (8)	165
C11—H11···F2^ii^	0.93	2.63	3.513 (8)	159
C10—H10*A*···O5^iii^	0.97	2.53	3.469 (11)	162
C15—H15···O5^iv^	0.93	2.55	3.345 (9)	144
C8—H8···*Cg*1^v^	0.93	2.82	3.740 (8)	173

Symmetry codes: (ii) *x*+1, *y*, *z*+1; (iii) −*x*+1, −*y*+1, −*z*+1; (iv) −*x*+1, −*y*, −*z*+1; (v) *x*, *y*+1, *z*.
